# *CYP2C19* Genotype is Associated with Citalopram Treatment Outcomes in a Real‐World Setting

**DOI:** 10.1002/cpt.70285

**Published:** 2026-04-14

**Authors:** Yoomi Park, Yitian Zhou, Volker M. Lauschke

**Affiliations:** ^1^ Department of Physiology and Pharmacology and Center for Molecular Medicine Karolinska Institutet and University Hospital Stockholm Sweden; ^2^ Medical Research Center Seoul National University College of Medicine Seoul South Korea; ^3^ Dr Margarete Fischer‐Bosch Institute of Clinical Pharmacology Stuttgart Germany; ^4^ University of Tübingen Tübingen Germany; ^5^ Department of Pharmacy, the Second Xiangya Hospital Central South University Changsha China

## Abstract

CYP2C19 metabolizes various selective serotonin reuptake inhibitors (SSRIs) and genetic *CYP2C19* variants are associated with SSRI tolerability and response. Yet, whether CYP2C19 variability also impacts citalopram response remained unclear. We here evaluated associations between *CYP2C19* genotypes and citalopram prescription data of 11,079 patients from the UK Biobank. Importantly, CYP2C19 ultrarapid metabolizers (UMs) were more likely to experience therapeutic failure (OR 2.03, *P* = 0.002) and both CYP2C19 poor metabolizers (PMs) and UMs exhibited an increased likelihood of treatment resistance (PMs, OR 1.85, *P* = 0.06; UMs, OR 1.74, *P* = 0.05). Furthermore, inferred CYP2C19 metabolizer status showed significant monotonic trends with citalopram maintenance dose (from 19.7 mg/d in PMs to 24.4 mg/d in UMs; Jonckheere–Terpstra *P* = 0.017) and time to dose escalation (2.5 years in PMs to 1.7 years in UMs; *P* = 0.006). Besides *CYP2C19*, rare variant analyses identified *BMP2K* as a novel candidate locus for SSRI response. *BMP2K* variants were significantly associated with therapeutic failure in citalopram patients (*P* = 5.49 × 10^−6^) and this association was replicated in 220 escitalopram users (*P* = 0.042). Furthermore, higher *BMP2K* variant burden significantly correlated with lower maintenance dose. These findings provide the first report of a U‐shaped association between CYP2C19 metabolizer status and citalopram therapeutic outcomes and pinpoint *BMP2K* as a novel candidate gene with potential contribution to interindividual differences in citalopram response. Collectively, these results demonstrate that *CYP2C19* genotype is significantly associated with citalopram outcomes in a real‐world setting in which patient treatment was not guided by pharmacogenetics, thus avoiding open‐label awareness, eliminating expectation bias of genetic testing and highlighting the value of large‐scale biobank data for advancing precision psychiatry.

**Study Highlights**

**WHAT IS THE CURRENT KNOWLEDGE ON THE TOPIC?**

*CYP2C19* genotype is known to influence plasma exposure and clinical response to escitalopram, but evidence for genetic effects on citalopram therapeutic outcomes has been limited.

**WHAT QUESTION DID THIS STUDY ADDRESS?**

This study examined how *CYP2C19* variation affects citalopram therapeutic failure, treatment resistance, and time to dose escalation using drug switching as proxy. In addition, novel genetic contributors for citalopram treatment response were explored.

**WHAT DOES THIS STUDY ADD TO OUR KNOWLEDGE?**

Both reduced and increased CYP2C19 activity were associated with higher switching rates. Ultrarapid metabolizers showed a reduced time to dose escalation. Variations in *BMP2K* were identified as an exploratory candidate linked to lower citalopram maintenance dose and significance was replicated in escitalopram users.

**HOW MIGHT THIS CHANGE CLINICAL PHARMACOLOGY AND TRANSLATIONAL SCIENCE?**

These findings show that *CYP2C19* genotype impacts citalopram response in a real‐world setting even when genetic information is unavailable, suggesting that *CYP2C19* variability should be considered to guide citalopram prescribing. Furthermore, these results demonstrate how large‐scale biobank data can inform precision psychiatry.

Selective serotonin reuptake inhibitors (SSRIs) such as citalopram are first‐line treatments for major depressive disorder (MDD) with well‐established efficacy.^1^ Despite their widespread use, treatment outcomes vary substantially between individuals, with only about one‐third of patients achieving remission after the first antidepressant trial.^2^, ^3^ A major contributor to this variability is interindividual difference in drug metabolism, efficacy, and tolerability, much of which is influenced by genetic factors.^4^, ^5^

Among the pharmacogenes implicated in SSRI metabolism, *CYP2C19* plays a central role in the biotransformation of citalopram, its S‐enantiomer escitalopram and sertraline.^6^ Carriers of decreased function alleles, such as CYP2C19*2, *3 or *4, exhibit higher plasma concentrations and an increased risk of adverse effects, whereas carriers of the increased‐function allele CYP2C19*17 display lower exposure and potentially reduced efficacy.^7^, ^8^ Several studies, including large real‐world analyses of escitalopram and sertraline users, have furthermore demonstrated significant associations between CYP2C19 metabolizer status and clinical outcomes.^9^, ^10^ However, corresponding evidence for citalopram remains limited and, at times, conflicting.^11^, ^12^, ^13^ Large‐scale biobanks that integrate longitudinal prescription data with genomic information provide new opportunities to evaluate pharmacogenetic effects under routine clinical conditions.^14^ Leveraging these resources enables the investigation of associations between genetic variation and diverse phenotypic endpoints that better capture prescribing behavior and therapeutic adjustment in real‐world settings.

Here, we leveraged genetic and electronic health record data from 11,079 patients with citalopram prescriptions in the UK Biobank to evaluate associations between therapeutic outcomes and *CYP2C19* genotypes. Specifically, we focused on patients diagnosed with MDD to examine associations between genotypes and switching‐based proxies of therapeutic failure, treatment resistance, and time to dose escalation. Furthermore, we conducted a genome‐wide rare variant burden analysis to identify novel genetic contributors to variability in citalopram maintenance dose and treatment response.

## METHODS

### Patient cohorts and genotype groups

Major depressive disorder (MDD) was defined from electronic healthcare records following the approach by Fabbri et al.^15^ In short, participants were classified as MDD if they had at least two primary care diagnostic codes for depression recorded in UK Biobank primary care data.^16^ Individuals with diagnostic codes for bipolar disorder, psychotic disorders, or substance‐use disorders were excluded to avoid diagnostic overlap. *CYP2C19* star alleles were grouped as follows: CYP2C19*2 (rs4244285), CYP2C19*3 (rs4986893/rs57081121), and CYP2C19*4 (rs28399504) were considered as loss‐of‐function alleles (Null), whereas CYP2C19*17 (rs12248560) was treated as gain‐of‐function. Because CYP2C19*17 is located in the promotor region and is not captured by exome sequencing, this variant was obtained from the UK Biobank genotyping‐imputation dataset, where it showed high imputation quality (MAF = 0.213; INFO = 0.998). All other variants were derived from the UK Biobank exome sequencing data. Metabolizer phenotypes were inferred from diplotypes as follows: Poor metabolizers (PM) = Null/Null; intermediate metabolizers (IM) = *1/Null or *17/Null; normal metabolizers (NM) = *1/*1 and ultrarapid metabolizers (UM) = *1/*17 or *17/*17.

### Study endpoints and statistical analysis

#### Drug switching

Antidepressant prescription records were obtained from the UK Biobank primary care linkage, which includes coded prescription events (drug codes such as British National Formulary [BNF] classifications and/or dm + d codes) together with the dates of issue. Drug names were standardized using a predefined mapping of generic and brand names. For each eligible MDD case, the first prescription of citalopram was identified as the index antidepressant exposure. Therapeutic failure was defined as a switch from the citalopram to any other antidepressant within 1 year after the last citalopram prescription as reported previously.^9^ Treatment‐resistant depression was defined as the occurrence of at least two distinct antidepressant switches,^15^ where switches were defined as initiation of a different antidepressant after a treatment episode of at least six consecutive weeks, with no more than 14 weeks between the end of one antidepressant and the start of the next, to distinguish genuine treatment change from discontinuation. Concomitant exposure to CYP2C19 inhibitors was assessed by identifying prescriptions for proton pump inhibitors (omeprazole, esomeprazole, lansoprazole, pantoprazole, and rabeprazole) and other CYP2C19 inhibitors (fluvoxamine, fluconazole, ketoconazole, ticlopidine) within ±30 days around the last citalopram prescription for therapeutic failure, and from 30 days before citalopram initiation to 30 days after treatment end for treatment resistance. Augmentation therapy was defined as prescriptions for atypical antipsychotics (aripiprazole, quetiapine, olanzapine, risperidone, paliperidone, lurasidone, and cariprazine), lithium, buspirone, ketamine/esketamine or mood stabilizers (valproate, lamotrigine or carbamazepine) within the same predefined exposure windows. Psychiatric comorbidity was defined based on ICD‐10 diagnostic codes recorded at any time in the medical history, including anxiety disorders (F40–F41, F43), obsessive‐compulsive disorder (F42) and self‐harm or suicide attempt (X60–X84, Y10–Y34). These variables were included as covariates in sensitivity analyses.

#### Maintenance dose

Primary care prescription records were used to derive individual citalopram maintenance doses. Prescriptions related to combination therapies or those missing dosage or quantity information were excluded. All prescriptions were normalized by converting dosage units to milligrams and computing the total prescribed amount per issue date. As prescription duration is not explicitly recorded, the average daily dose was estimated from the interval between consecutive prescriptions, calculated as the prescribed strength multiplied by the dispensed quantity and divided by the number of days until the subsequent prescription.^17^ For each participant, maintenance dose was defined as the mean daily dose across the five most recent valid prescription intervals to capture stable dosing while minimizing early dose titration. Participants with fewer than five valid prescription intervals were excluded from the analysis. Monotonic trends in maintenance dose across *CYP2C19* genotype and metabolizer groups were evaluated using the Jonckheere–Terpstra test.

#### Dose escalation

Dose escalation was assessed using longitudinal citalopram prescription records. For each participant, dose strength (mg) was extracted from the drug name or quantity field using regular expressions, and prescriptions issued on the same day were aggregated to obtain a single daily dose. For each individual, prescriptions were chronologically ordered, and changes in dose between consecutive prescription dates were calculated. Dose escalation, defined as any increase in dose between two consecutive prescriptions, served as the primary endpoint. The first citalopram prescription date was taken as baseline, and follow‐up continued until the first escalation event or the last recorded citalopram prescription, whichever came first. Participants were included if they had at least one citalopram prescription and an available *CYP2C19* genotype or diplotype assignment. Escalation frequencies were compared across *CYP2C19* genotype groups using Fisher's exact tests, and the time to first escalation was analyzed using Kaplan–Meier estimators, log‐rank tests, and Cox proportional hazards models with *1/*1 as the reference group. Ordinal trend effects across metabolizer categories were evaluated using logistic regression and an ordinal Cox model. As all analyses of CYP2C19 and citalopram response outcomes were hypothesis‐driven based on prior pharmacogenetic evidence, no adjustment for multiple testing was applied.

### Gene‐based rare variant association analysis

Variant annotation was performed using ANNOVAR,^18^ including gene assignment, functional consequence, and population allele frequencies based on the gnomAD Non‐Finnish European (NFE) reference data. Variants were filtered to retain rare (variants with minor allele frequency [MAF] < 5% in gnomAD NFE) nonsynonymous, stop‐gain/loss or splice‐site variants. For CYP2C19, the well‐established increased‐function variant CYP2C19*17 (rs12248560) was included in the gene‐level tests to ensure comprehensive representation of functional alleles. Gene‐level association tests were performed for both therapeutic failure and treatment‐resistant depression endpoints using the burden, SKAT and SKAT‐O methods implemented in the SKAT R package.^19^ All models were adjusted for age, sex, and the first 10 genetic principal components to account for population stratification. Statistical significance was defined as Benjamini–Hochberg false discovery rate *q* < 0.1. To evaluate cross‐drug concordance, we extracted individuals with a history of escitalopram use and applied the same endpoints and analytical pipelines. Genes showing significant replication in the escitalopram cohort (*p* < 0.05) were considered consistent candidates. Associations between rare variant burden and average daily citalopram maintenance dose were calculated by considering each participant included in the gene‐based analysis. Participants were binned into 0, 1, or ≥2 rare variants per gene and associations with daily citalopram doses were assessed using the Jonckheere–Terpstra trend test, both in the full cohort and in the *CYP2C19* normal metabolizer subset (*1/*1). All analyses were conducted in R (v3.6.3).

## RESULTS

### Real‐world association of *CYP2C19* variability and citalopram therapeutic response

**Figure 1:**
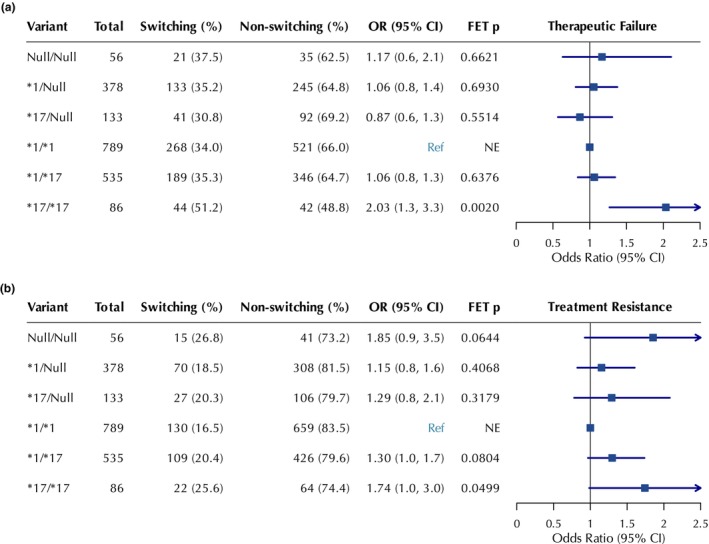
Impact of *CYP2C19* on citalopram therapeutic failure and treatment resistance in major depressive disorder (MDD). UK Biobank participants with MDD (≥2 depression diagnosis codes; exclusions for bipolar, psychotic, and substance‐use disorders) who had a history of citalopram use were stratified by *CYP2C19* diplotype. Panels show the risk of (**a**) therapeutic failure and (**b**) treatment resistance. Odds ratios (OR) and confidence intervals (ci) are shown relative to the *1/*1 group. For visualization purposes, the x‐axis was truncated at OR = 2.5, and confidence intervals exceeding this limit are indicated with arrows. FET = Fisher's exact test.

Among 11,079 participants with a history of citalopram use in the UK Biobank, 7,637 (69%) had a least one recorded depression diagnosis code, of whom 2,200 (28.8%) met the definition of MDD (≥2 distinct depression diagnosis codes). After excluding individuals with bipolar disorder, psychotic disorder, or substance‐use disorder, 1,977 participants remained in the final MDD cohort. To evaluate the impact of *CYP2C19* genotype on real‐world citalopram treatment outcomes, we first compared the frequency of drug switching across metabolizer subgroups among patients diagnosed with MDD. For therapeutic failure, UMs (*17/*17) exhibited a significantly increased risk of switching (OR = 2.03, 95% CI: 1.3–3.3; *P* = 0.002; **Figure** **1** **a**). PMs (Null/Null) did not show a significant increase but showed a trend toward increased citalopram therapeutic failure (OR = 1.17, 95% CI 0.6–2.1; *P* = 0.66). A similar pattern was observed for treatment resistance, where both *17/*17 (OR = 1.74, 95% CI: 1.0–3.0; *P* = 0.0499) and Null/Null (OR = 1.85, 95% CI: 0.9–3.5; *P* = 0.064) genotypes were associated with elevated odds of switching (**Figure** **1** **b**). These findings remained robust after adjustment for potential covariates (**Table** **S1**) and in analyses stratified by metabolizer phenotype (**Figure** **S1**). The observed patterns suggest that UMs may be more likely to switch medications due to subtherapeutic exposure, whereas reduced hepatic clearance in PMs may lead to overexposure and toxicity. Indeed, when examining the association between *CYP2C19* genotype and hyponatremia, a recognized adverse reaction to SSRIs,^20^ a significant increase in risk among CYP2C19 PMs was observed (*P* = 0.044), consistent with systematically higher exposure (**Figure** **S2**). In line with these findings, CYP2C19 PMs also exhibited the highest proportion of between‐class and the lowest proportion of within‐class antidepressant switching (**Figure** **S3**), further supporting potential treatment intolerance. Together, these findings reveal a U‐shaped distribution across *CYP2C19* diplotypes, with both PMs and UMs showing higher switching rates than IMs and NMs in routine clinical practice.

### Association of CYP2C19 status with citalopram maintenance dose

**Figure 2:**
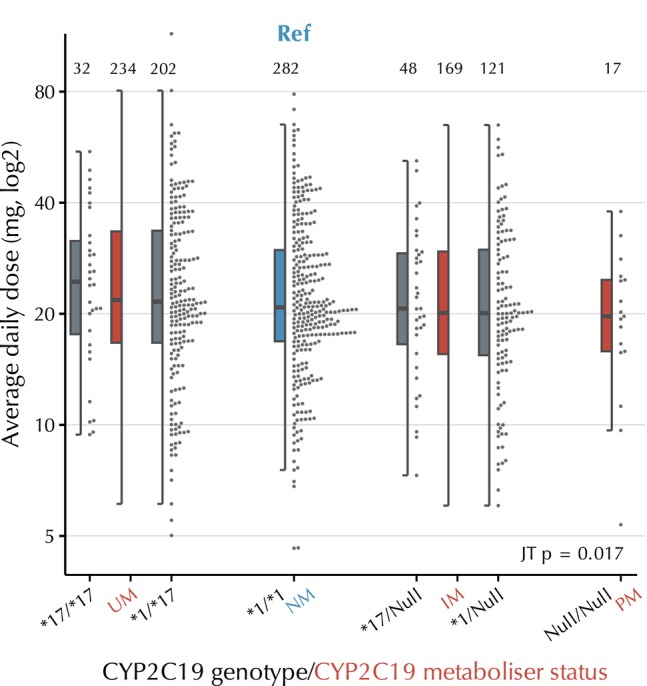
Association between CYP2C19 diplotype, metabolizer status and average maintenance dose of citalopram. Each dot represents an individual's prescribed maintenance dose (mg/day). Error bars show the interquartile ranges, with the median indicated by a horizontal line. Genotypes are presented alongside their corresponding metabolizer phenotypes (UM, NM, IM, PM) in grouped panels, with *1/*1 as the reference (Ref). Numbers above each group indicate the sample size for the corresponding *CYP2C19* diplotype. The displayed p‐value indicates the Jonckheere–Terpstra (JT) test for a monotonic trend in maintenance dose across metabolizer groups.

To examine whether CYP2C19 metabolic activity influences real‐world citalopram dosing, average maintenance doses were compared across inferred metabolizer phenotypes in participants with MDD (**Figure** **2**). Median maintenance doses were consistently graded across metabolizer categories. Individuals carrying CYP2C19*17/*17 had the highest median dose (24.4 mg/d), with a gradual decrease toward lower activity groups (*1/*17 = 21.6 mg/d; *1/*1 = 20.8 mg/d; *17/Null = 20.7 mg/d; *1/Null = 20.1 mg/d; Null/Null = 19.7 mg/d). Combined, UMs (*17/*17 and *1/*17) tended to receive significantly higher doses than NMs (median 21.8 mg/day compared with 20.7 mg/d in NMs), whereas PMs were prescribed lower doses than the reference group (median 19.7 mg/day). A significant monotonic trend was observed across metabolizer categories (Jonckheere–Terpstra *P* = 0.017). This pattern was consistent when alternative definitions of maintenance dose (i.e., the two most recent prescription intervals) were used (**Table** **S2**). These results demonstrate that average daily maintenance doses increased with increasing CYP2C19 metabolism, consistent with hepatic clearance driving higher dose requirements.

### *CYP2C19* genotype and dose escalation patterns among citalopram users

**Figure 3:**
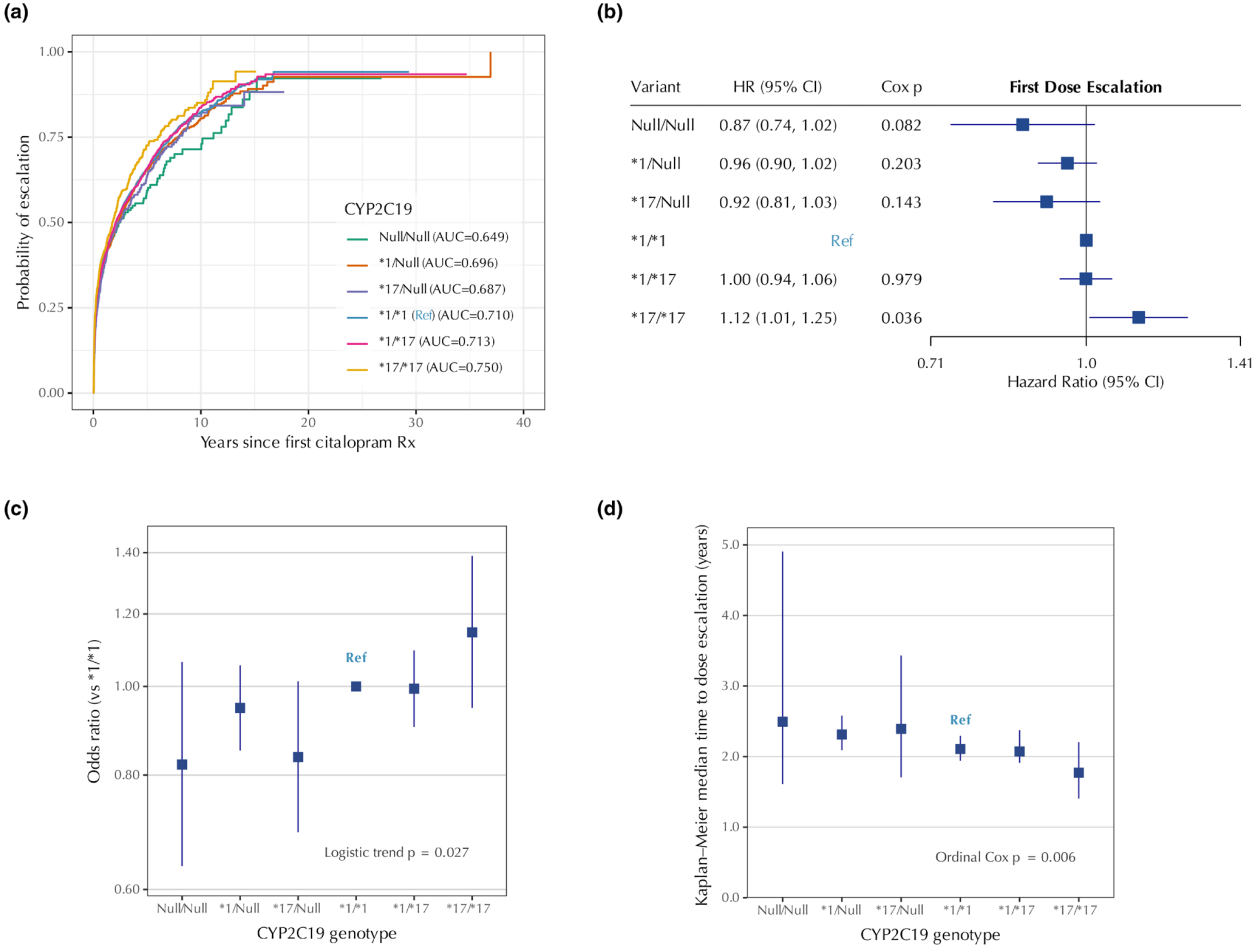
*CYP2C19* genotype and citalopram dose escalation. (**a**) Kaplan–Meier plots showing the cumulative probability of first dose escalation, stratified by *CYP2C19* diplotype. Participants were censored at last observed prescription date. (**b**) Cox proportional hazards model for time to first dose escalation, with hazard ratios (HR) relative to *1/*1 and 95% confidence intervals (ci). (**c**) Odds ratios for any dose escalation event while on citalopram, relative to *1/*1; error bars indicate 95% CI. The displayed p‐value is from a logistic regression modeling a monotonic trend across metabolizer status. (**d**) Kaplan–Meier median time to first dose escalation by diplotype.; error bars indicate 95% CI. The displayed p‐value stems from a Cox proportional hazards model including an ordinal metabolizer term.

We next evaluated whether *CYP2C19* genotype influenced the likelihood or timing of citalopram dose escalation. Overall, the CYP2C19*17/*17 subgroup showed a slightly higher cumulative probability of dose escalation compared with other diplotypes, whereas Null/Null carriers tended to have a lower probability (**Figure** **3** **a**). In the Cox proportional hazards model, *17/*17 carriers exhibited a modestly increased likelihood of dose escalation (HR = 1.12, 95% CI 1.01–1.25; *P* = 0.036), with a gradual decrease in hazard ratio toward lower activity groups (**Figure** **3** **b**). These finding remained consistent after adjusting for a broad range of psychiatric indications, including off‐label use (**Table** **S3**). Logistic regression analyses of any escalation event yielded consistent results, showing a significant monotonic trend across metabolizer groups (**Figure** **3** **c**, *P* = 0.027). Median time to first dose escalation also varied by genotype (**Figure** **3** **d**); *17/*17 carriers had the shortest median time (1.77 years), whereas *1/*1 and lower activity diplotypes exhibited progressively longer times to escalation (*1/*17 = 2.07 years; *1/*1 = 2.11 years; *1/Null = 2.32 years; Null/Null = 2.5 years Jonckheere–Terpstra trend *P* = 0.006). Collectively, these findings indicate that CYP2C19 UMs are more likely to require citalopram dose escalations and that these are required earlier in the clinical treatment regimen.

### Genome‐wide association analysis of citalopram response

To identify novel genetic determinants of citalopram response beyond *CYP2C19*, we conducted a rare variant association analysis using exome sequencing data from patients with MDD who had received citalopram. Gene‐based rare variant association tests identified two candidate genes showing significant associations with citalopram response after FDR correction (*q* < 0.1; **Table** **1**). In participants treated with citalopram, *BMP2K* was significantly associated with therapeutic failure (burden *P* = 5.49 × 10^−6^; SKAT‐O *P* = 1.5 × 10^−5^), while *METTL18* demonstrated a significant signal for treatment resistance (burden *P* = 2.41 × 10^−6^; SKAT‐O *P* = 8.98 × 10^−6^). After additional adjustment for clinical covariates including psychiatric comorbidity, augmentation therapy and CYP2C19 inhibitor exposure, the *BMP2K* signal remained robust, whereas the *METTL18* association did not remain significant after multiple testing correction (**Table** **S4**). To validate these signals, we assessed the same genes across related SSRIs. In escitalopram users (*n* = 220), *METTL18* did not replicate; however, importantly, the association with *BMP2K* was confirmed in this independent replication cohort (burden *P* = 0.0428). The signal was overall distributed across 44 variants of which rs2288255, rs2114202, and rs143310663 showed the strongest signals (**Figure** **S4**). Functional annotation using multiple *in silico* prediction tools indicated that 61.4% (27/44) of variants were predicted to be deleterious by the majority of tools, supporting their potential biological relevance (**Table** **S5**).

**Table 1:** Gene‐based rare variant association tests for therapeutic failure and treatment resistance. Rare variants (gnomAD minor allele frequencies in Non‐Finnish Europeans <5%) were included. For citalopram users, significant associations after multiple testing correction are reported from burden, SKAT, and SKAT‐O tests. To assess concordance across closely related SSRIs, the candidate genes were also evaluated in escitalopram users.

Gene	N variants	Therapeutic failure			Treatment‐resistant		
		Burden	SKAT	SKAT‐O	Burden	SKAT	SKAT‐O
Citalopram							
*BMP2K*	44	**5.49E‐06**	**0.0021**	**1.50E‐05**	0.0899	0.3328	0.1615
*METTL18*	10	**0.0208**	0.0532	**0.0324**	**2.41E‐06**	**0.0003**	**8.98E‐06**
Escitalopram							
*BMP2K*	12	**0.0428**	0.4132	0.0743	0.6973	0.4876	0.6763
*METTL18*	3	0.7760	0.8793	1	0.5961	0.8772	0.7861

*Note:* Bold indicates *p* values below 0.05.

**Figure 4:**
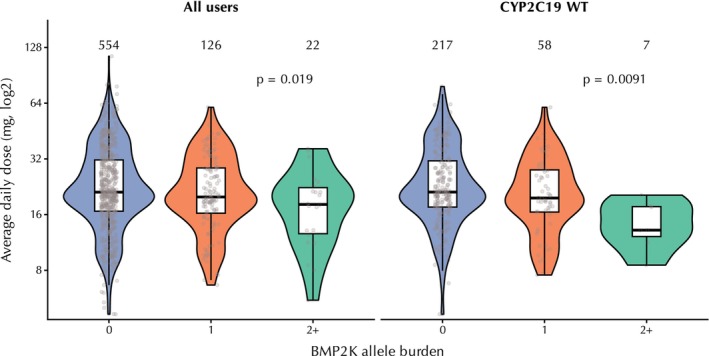
*BMP2K* variant burden and citalopram maintenance dose. The distribution of average daily citalopram dose (mg/day) is shown by *BMP2K* rare‐allele burden category (0, 1, or 2+ alleles; Left: all users; right: *CYP2C19* wild‐type (*1/*1) users only). Dots represent individual participants. Sample sizes are shown above each group. The y‐axis is displayed on a log2‐transformed scale. Jonckheere–Terpstra P‐values indicate dose trends across burden categories.

To further explore the functional relevance of these findings, we examined whether *BMP2K* variant burden correlated with average daily citalopram dose. A significant trend was observed across allele burden categories (Jonckheere–Terpstra *P* = 0.019), with higher *BMP2K* burden associating with lower maintenance dose (**Figure** **4**). Interestingly, this association appeared stronger when analyses were restricted to CYP2C19 NMs (*P* = 0.0091), suggesting that rare *BMP2K* variation might contribute independently to interindividual variability in citalopram response. Together, these analyses highlight *BMP2K* as a candidate gene of interest that may influence SSRI response independently of CYP2C19‐mediated effects on drug disposition. We note however that given the absence of an independent replication cohort, these results should for now be interpreted with caution.

## DISCUSSION

In this study, we leveraged real‐world prescribing and genomic data from the UK Biobank to evaluate genetic factors impacting citalopram response. This integration of longitudinal prescription records with genomic profiles in large population cohorts provides a powerful framework to identify both established and previously unrecognized contributors to antidepressant treatment variability. Our analyses reveal a U‐shaped relationship between CYP2C19 metabolizer status and switching‐based proxies of therapeutic failure, with both poor and ultrarapid metabolizers (UMs) showing higher switching rates than normal and intermediate metabolizers. This pattern may reflect distinct pharmacological mechanisms. In UMs, increased hepatic clearance may result in lower drug exposure that could contribute to reduced treatment effectiveness, whereas plasma levels in PMs may be higher and potentially associated with increased adverse effects and intolerance. The gradient in maintenance dose across metabolizer groups supports this interpretation. Similar association patterns have previously been reported for escitalopram and sertraline^9^, ^10^; however, we are not aware of similar reports for citalopram. Importantly, unlike in dedicated pharmacogenomic trials,^21^ the observed differences between metabolizer groups cannot be accounted for by placebo effects, thus providing a methodologically clean approach that reveals the impact of genetic factors on pharmacological phenotypes.

Because *CYP2C19* genotyping is not routinely implemented in UK psychiatric care, the observed dose patterns probably reflect empirical adjustments by clinicians rather than genotype‐guided prescribing. The consistency between genotype, clinical titration behavior, and prescription‐based therapeutic outcome measures supports the translational relevance of *CYP2C19* as a biomarker for SSRI optimization in real‐world care. Ultrarapid metabolizers also demonstrated a greater likelihood and earlier onset of dose escalation during citalopram treatment, suggesting a reproducible pharmacogenetic influence on longitudinal prescribing behavior. Lastly, a significantly higher incidence of hyponatremia in PMs suggests that CYP2C19 activity can impact both efficacy‐ and safety‐related outcomes. On an absolute scale, UMs showed a ~17% higher switching risk for therapeutic failure compared with NMs (51.2% vs 34.0%), corresponding to ~17 additional switching events per 100 citalopram‐treated patients. This corresponds to an approximate number needed to test (NNT) of 6, that is, preemptive genotyping would identify one patient who would eventually switch medication due to therapeutic failure for every six patients tested. For treatment resistance, differences were smaller (~9% for UMs and ~10% for PMs, corresponding to NNTs of ~11 and ~10, respectively). While effects sizes varied across outcomes, these results suggest that *CYP2C19* genotype may help identify patients at increased risk of treatment failure in real‐world clinical settings.

Switching frequencies in our dataset were slightly higher than frequencies reported in the literature. In 2,087 patients treated with escitalopram in Norway, switching occurred in 20.4% of CYP2C19 UMs (*1/*17 and *17/*17) and 30.7% of PMs.^9^ Using the same definitions, we observed therapeutic failure in 37.5% of UMs and 37.5% of PMs, suggesting that these differences may reflect a higher sensitivity of UMs and PMs to citalopram compared with escitalopram. Importantly, both studies demonstrated increased switching frequencies among poor and UMs, supporting the reproducibility of CYP2C19‐related SSRI response in European populations. Compared with escitalopram users in Chinese Han patients,^10^ our switching rates were similar for normal (16.5% vs. 13.6% in Han) and intermediate metabolizers (19% vs. 24.6% in Han). Interestingly, in this Chinese cohort, switching frequencies were higher in UMs compared with PMs (66.7% in UMs vs. 31% in PMs), whereas the pattern was inverse and overall lower in UK biobank data (21.1% in UMs and 26.8% in PMs). Notably, while the studies from Norway and China were conducted in a controlled hospital setting at a single site, data from the UK Biobank reflects real‐world treatment practices in a national healthcare setting. Thus, while there are minor quantitative differences, our results demonstrate that increased switching among both poor and ultrarapid CYP2C19 metabolizers even occurs “naturally” in response to patient intolerability or lack of response, even when patient genotype is not explicitly considered.

Beyond *CYP2C19*, gene‐based rare variant analyses identified two exploratory loci, *METTL18* and *BMP2K*, associated with citalopram response of which the latter replicated in escitalopram users. *METTL18* encodes an N‐terminal histidine methyltransferase implicated in ribosomal function and translational regulation, processes linked to synaptic plasticity and antidepressant response.^22^ Despite this biological plausibility, the lack of replication warrants caution. The escitalopram cohort was substantially smaller (*n* = 220), limiting power for rare variant analyses. Moreover, the *METTL18* signal was driven by a small number of variants (*n* = 10), increasing susceptibility to stochastic effects, and it did not remain significant after covariate adjustment, suggesting potential confounding. Collectively, these findings position *METTL18* as an exploratory candidate and its association with citalopram should be interpreted cautiously in the absence of further external replication. BMP2K encodes a serine/threonine kinase linked to the bone morphogenetic protein (BMP) signaling network. While *BMP2K* itself has not previously been implicated in antidepressant response, hippocampal BMP signaling has been shown to regulate adult neurogenesis and mediate behavioral effects of antidepressants,^23^ providing biological plausibility. Notably, the *BMP2K* pattern, where nonswitchers more frequently carried rare *BMP2K* variants and those with higher variant burden required lower citalopram doses, did not mirror the U‐shaped profile observed for *CYP2C19*. This difference is plausible given their distinct biological roles: while CYP2C19 impacts citalopram pharmacokinetic, where both reduced and increased metabolism can result in suboptimal exposure, *BMP2K* likely reflects pharmacodynamic processes. Variants in *BMP2K* may therefore confer greater antidepressant sensitivity, enabling effective symptom control at lower doses, particularly among CYP2C19 NMs. Given the exploratory nature of these analyses and the limited replication, however, we want to emphasize that these associations should be interpreted cautiously. Nevertheless, they do carefully suggest a picture in which complementary pharmacokinetic (*CYP2C19*) and pharmacodynamic (*BMP2K*) mechanisms can act in concert to influence real‐world citalopram response.

A notable limitation of the present study is that treatment outcomes were inferred from prescription records rather than from direct clinical assessments. Such data do not ensure that patient adherence, which may fluctuate particularly in individuals with MDD. Accordingly, the endpoints analyzed here, including switching behavior, dose escalation and maintenance dosing, should be interpreted as proxies reflecting real‐world prescribing patterns and clinical decision‐making rather than direct measures of pharmacological efficacy or drug exposure. In addition, prescription‐based endpoints cannot distinguish the underlying reasons for treatment changes, such as adverse effects versus lack of efficacy. Although we adjusted for several clinical factors in sensitivity analyses, residual confounding from unmeasured (e.g., symptom severity) or incompletely captured variables (e.g., comorbid anxiety in MDD patients or over‐the‐counter CYP2C19 inhibitors) cannot be excluded. Nevertheless, despite the inherent noise introduced by variable adherence, such measures are widely used in pharmacoepidemiological studies and enable the study of treatment trajectories in large cohorts under routine clinical conditions. Finally, the relatively small number of CYP2C19 PMs limited statistical power for subgroup analyses; thus, nonsignificant findings in this group should be interpreted with caution, as modest associations may have gone undetected.

In summary, this study provides real‐world evidence linking CYP2C19 genotype to treatment adjustments during citalopram therapy and identifies novel loci of interest that may contribute to interindividual differences in antidepressant treatment outcomes. These findings support the continued integration of pharmacogenomic data into clinical and real‐world settings to refine precision prescribing of antidepressants.

## FUNDING

The authors' laboratory received funding from the Swedish Research Council [grant numbers: 2021–02801, 2023–03015 and 2024–03401], by the SciLifeLab and Wallenberg National Program for Data‐Driven Life Science [WASPDDLS22:006], the Robert Bosch Foundation, Stuttgart, Germany, and the National Research Foundation of Korea (NRF) grant funded by the Korea government (MSIT) (RS‐2023‐00209528). This research has been conducted using the UK Biobank Resource under application number 354002.

## CONFLICT OF INTEREST

VML is co‐founder, CEO and shareholder of HepaPredict AB, as well as co‐founder and shareholder of Shanghai Hepo Biotechnology Ltd. All other authors declare that they have no competing interests.

## AUTHOR CONTRIBUTIONS

Y.P., Y.Z. and V.M.L. designed the research. Y.P. performed the research. Y.P., Y.Z., and V.M.L analyzed the data. Y.P. and V.M.L. wrote the manuscript.

## Supporting information


Table S1.

Figure S1.
