# Supplementary material for: CYP2C19 Genotype is Associated with Citalopram Treatment Outcomes in a Real‐World Setting
Source: Clin Pharmacol Ther. 2026 Apr 14;120(2):412–9. doi: 10.1002/cpt.70285 (PMC13339076; doi:10.1002/cpt.70285)
Supplement: Supplementary file 1 — Table S1. [file CPT-120-412-s001.docx]

**Supplementary Table S1. Impact of CYP2C19 on therapeutic failure and treatment resistance to citalopram in major depressive disorder (MDD).** UK Biobank participants with MDD (≥2 depression diagnosis codes; exclusions for bipolar, psychotic, and substance-use disorders) who had a history of citalopram use were stratified by CYP2C19 diplotype. Panels show the risk of (A) therapeutic failure and (B) treatment resistance within a 1-year follow-up window. Switching percentages represent the proportion of individuals who switched antidepressants within each diplotype group. The *1/*1 diplotype was used as the reference group.

**(A) Therapeutic Failure**

| CYP2C19 | Model 1: Adjusted | | | | | Model 2: PPI-excluded | | |
| --- | --- | --- | --- | --- | --- | --- | --- | --- |
|  | Total (N=1,977) | Switching (%) | OR  (95% CI) | P | Total (N=1,697) | Switching (%) | OR  (95% CI) | P |
| Null/Null | 56 | 21 (37.5%) | 1.13 (0.6-2.0) | 0.673 | 51 | 19 (37.3%) | 1.21 (0.7-2.2) | 0.520 |
| *1/Null | 378 | 133 (35.2%) | 1.07 (0.8-1.4) | 0.599 | 325 | 112 (34.5%) | 1.11 (0.8-1.5) | 0.461 |
| *17/Null | 133 | 41 (30.8%) | 0.88 (0.6-1.3) | 0.520 | 117 | 38 (32.5%) | 1.01 (0.7-1.5) | 0.960 |
| *1/*1 | 789 | 268 (34.0%) | Ref | - | 679 | 220 (32.4%) | Ref | - |
| *1/*17 | 535 | 189 (35.3%) | 1.08 (0.9-1.4) | 0.523 | 450 | 160 (35.6%) | 1.18 (0.9-1.5) | 0.209 |
| *17/*17 | 86 | 44 (51.2%) | 2.11 (1.3-3.3) | 0.001 | 75 | 38 (50.7%) | 2.19 (1.4-3.6) | 0.001 |

**(B) Treatment Resistance**

| CYP2C19 | Model 1: Adjusted | | | | | Model 2: PPI-excluded | | |
| --- | --- | --- | --- | --- | --- | --- | --- | --- |
|  | Total (N=1,977) | Switching (%) | OR  (95% CI) | P | Total (N=1,551) | Switching (%) | OR  (95% CI) | P |
| Null/Null | 56 | 15 (26.8%) | 1.81 (0.9-3.3) | 0.062 | 45 | 13 (28.9%) | 2.19 (1.1-4.2) | 0.025 |
| *1/Null | 378 | 70 (18.5%) | 1.18 (0.9-1.6) | 0.317 | 302 | 55 (18.2%) | 1.24 (0.9-1.8) | 0.248 |
| *17/Null | 133 | 27 (20.3%) | 1.32 (0.8-2.1) | 0.242 | 105 | 21 (20.0%) | 1.40 (0.8-2.3) | 0.213 |
| *1/*1 | 789 | 130 (16.5%) | Ref | - | 627 | 97 (15.5%) | Ref | - |
| *1/*17 | 535 | 109 (20.4%) | 1.33 (1.0-1.8) | 0.051 | 407 | 85 (20.9%) | 1.49 (1.1-2.1) | 0.016 |
| *17/*17 | 86 | 22 (25.6%) | 1.80 (1.0-3.0) | 0.027 | 65 | 14 (21.5%) | 1.53 (0.8-2.8) | 0.186 |

Associations were estimated using logistic regression. Model 1 includes all citalopram users and is adjusted for augmentation therapy, proton pump inhibitor (PPI) use, and psychiatric comorbidity (i.e., anxiety disorders, OCD, or self-harm diagnoses). Model 2 excludes individuals receiving PPIs and is adjusted for augmentation therapy and psychiatric comorbidity. Individuals with bipolar or psychotic disorders were excluded during cohort definition and were therefore not included as covariates.

**Supplementary Table S2. Comparison of maintenance dose using alternative prescription-based definitions.** Citalopram maintenance dose estimates across CYP2C19 genotype groups using three approaches: (i) the strength of the most recent prescription, (ii) the mean daily dose estimated from the two most recent valid prescription intervals, and (iii) the mean daily dose estimated from the five most recent valid prescription intervals (primary analysis). Interval-based maintenance dose (ii and iii) were calculated as the dispensed dose divided by the time between consecutive prescriptions. Doses based only on the most recent prescription strength (i) do not account for prescription quantity or interval length and therefore may reflect initial dose or early titration rather than stabilized maintenance dose.

|  | **Last prescription strength** | | | **Maintenance dose**  **(last 2 intervals)** | | | **Maintenance dose**  **(last 5 intervals)** | | |
| --- | --- | --- | --- | --- | --- | --- | --- | --- | --- |
| **Variant** | **n** | **mean** | **sd** | **n** | **mean** | **sd** | **n** | **mean** | **sd** |
| *17/*17 | 86 | 18.5 | 8.3 | 44 | 26.5 | 16.4 | 32 | 26.2 | 12.2 |
| UM | 620 | 18.1 | 8.2 | 302 | 27.3 | 28.3 | 234 | 26.0 | 14.5 |
| *1/*17 | 534 | 18.1 | 8.2 | 258 | 27.4 | 29.9 | 202 | 25.9 | 14.8 |
| NM | 789 | 18.0 | 7.9 | 364 | 23.5 | 21.2 | 282 | 24.5 | 12.4 |
| *17/Null | 133 | 18.5 | 7.9 | 63 | 22.4 | 15.2 | 48 | 23.0 | 10.7 |
| IM | 511 | 18.1 | 7.9 | 231 | 22.4 | 16.2 | 169 | 23.1 | 11.4 |
| *1/Null | 378 | 17.9 | 7.9 | 168 | 22.4 | 16.7 | 121 | 23.1 | 11.8 |
| PM | 56 | 18.9 | 8.7 | 21 | 21.9 | 12.3 | 17 | 20.4 | 8.15 |

**Supplementary Table S3. Cox proportional hazards model for time to first dose escalation, with hazard ratios relative to *1/*1.** Models were adjusted for any recorded depression diagnosis (≥1 diagnosis code) and psychiatric comorbidities, including bipolar disorder, psychosis, and other psychiatric conditions (i.e., anxiety disorders, OCD, and self-harm/suicide attempt).

| Variant | Total (N=11,079) | Any escalation (%) | HR (95% CI) | Cox p |
| --- | --- | --- | --- | --- |
| Null/Null | 272 | 159 (58.5) | 0.86 (0.74-1.01) | 0.071 |
| *1/Null | 2,174 | 1,345 (61.9) | 0.96 (0.90-1.02) | 0.179 |
| *17/Null | 516 | 304 (58.9) | 0.91 (0.81-1.03) | 0.131 |
| *1/*1 | 4,506 | 2,845 (63.1) | Ref |  |
| *1/*17 | 3,060 | 1,928 (63.0) | 1.00 (0.94-1.06) | 0.946 |
| *17/*17 | 551 | 365 (66.2) | 1.13 (1.01-1.26) | 0.028 |
| Depression | 7,637 | 5,066 (66.3) | 1.14 (1.08-1.20) | < 0.001 |
| Other psychic | 2,561 | 1,763 (68.8) | 1.08 (1.02-1.14) | 0.009 |
| Biopolar | 128 | 90 (70.3) | 0.98 (0.79-1.21) | 0.859 |
| Psychosis | 72 | 51 (70.8) | 0.99 (0.74-1.32) | 0.942 |

**Supplementary Table S4. Sensitivity analysis of gene-based rare variant associations with therapeutic failure and treatment resistance after adjustment for clinical covariates.** Models were adjusted for age, sex, the first ten principal components, psychiatric comorbidity, concomitant augmentation medications, and CYP2C19 inhibitor exposure. Results are shown separately for citalopram and escitalopram cohorts using burden, SKAT, and SKAT-O tests.

| Gene | N variants | Therapeutic Failure | | | Treatment Resistant | | |
| --- | --- | --- | --- | --- | --- | --- | --- |
|  |  | Burden | SKAT | SKAT-O | Burden | SKAT | SKAT-O |
| Citalopram | | | | | | | |
| *CYP2C19* | 31 | 0.2985 | 0.9393 | 0.4615 | 0.0712 | 0.0483 | 0.0781 |
| *BMP2K* | 44 | **3.06E-06** | **0.0039** | **9.72E-06** | 0.0900 | 0.3260 | 0.1618 |
| *METTL18* | 10 | **0.0299** | 0.0896 | **0.0496** | **7.15E-06** | **0.0004** | **2.39E-05** |
| Escitalopram | | | | | | | |
| *CYP2C19* | 11 | 0.4899 | 0.0647 | 0.1125 | 0.0749 | 0.5069 | 0.1308 |
| *BMP2K* | 12 | 0.0537 | 0.3849 | 0.0928 | 1 | 0.3421 | 0.5069 |
| *METTL18* | 3 | 0.7752 | 0.8752 | 1 | 0.5779 | 0.7263 | 0.7503 |

**Supplementary Table S5. In silico pathogenicity predictions for rare BMP2K variants included in the gene-based analysis.** Functional impact was assessed using nine in silico prediction tools. For categorical predictors, variants were classified as damaging (D) or neutral/tolerated (N) according to the original tool annotations. For score-based predictors, damaging thresholds were defined as REVEL > 0.5 and CADD > 20. Variants were classified as overall predicted damaging when more than half of the available predictors indicated a damaging effect.

| Variant ID | Variant type | gnomad AF | SIFT | Polyphen2 | REVEL | CADD | MutationTaster | PROVEAN | AlphaMissense | M-CAP | fathmm-XF coding | N tools damaging / available tools | Overall predicted damaging |
| --- | --- | --- | --- | --- | --- | --- | --- | --- | --- | --- | --- | --- | --- |
| rs772334759 | missense | 2.00E-04 | N | N | N | N | N | N | N | NA | N | 0/8 | N |
| rs765379124 | missense | 5.00E-04 | N | D | N | D | D | N | D | NA | D | 5/8 | D |
| rs149914551 | missense | 0.0235 | D | D | D | D | D | D | D | NA | D | 8/8 | D |
| rs770167074 | missense | 2.63E-05 | D | D | N | D | D | D | D | D | D | 8/9 | D |
| rs201553990 | missense | 1.00E-04 | N | D | N | N | D | D | N | D | D | 5/9 | D |
| rs760103775 | missense | NA | N | D | N | D | D | N | N | N | N | 3/9 | N |
| 4:78833583 A>G | missense | NA | N | D | N | D | D | N | N | D | D | 5/9 | D |
| rs1375384213 | missense | NA | N | N | N | D | D | D | D | D | D | 6/9 | D |
| rs765768439 | missense | 6.58E-06 | N | D | N | N | D | N | N | D | D | 4/9 | N |
| rs56143363 | missense | 4.00E-04 | N | N | D | D | D | D | N | D | D | 6/9 | D |
| rs139850341 | missense | 1.00E-04 | N | D | N | D | D | N | N | D | D | 5/9 | D |
| rs113647501 | missense | 2.00E-04 | D | D | N | D | D | D | N | D | N | 6/9 | D |
| rs926898798 | missense | 6.58E-06 | N | D | D | D | D | D | D | N | D | 7/9 | D |
| rs775795262 | missense | 6.58E-06 | D | D | D | D | D | D | N | D | N | 7/9 | D |
| rs147699895 | missense | 3.00E-04 | N | N | N | D | D | N | N | D | D | 4/9 | N |
| rs767760300 | missense | 1.31E-05 | N | N | N | N | N | N | N | N | N | 0/9 | N |
| rs2288255 | missense | 0.0411 | N | D | N | N | N | N | N | NA | N | 1/8 | N |
| rs374800739 | missense | 6.71E-06 | N | N | N | N | N | N | N | N | N | 0/9 | N |
| rs2114202 | missense | 0.0183 | N | N | N | N | N | N | N | NA | N | 0/8 | N |
| rs200441916 | missense | 0.002 | N | D | N | N | N | N | N | NA | N | 1/8 | N |
| rs1248523170 | missense | NA | D | N | N | D | N | N | N | D | N | 3/9 | N |
| 4:78871888 A>C | missense | NA | D | D | N | N | D | D | N | D | N | 5/9 | D |
| rs1323717443 | missense | 6.57E-06 | D | D | N | N | D | D | N | D | N | 5/9 | D |
| 4:78872624 A>G | missense | NA | D | N | N | D | D | D | N | D | N | 5/9 | D |
| rs367968961 | missense | 5.26E-05 | D | D | N | D | D | D | D | N | D | 7/9 | D |
| rs200263125 | missense | 8.00E-04 | D | D | N | D | D | D | N | N | D | 6/9 | D |
| rs182929460 | missense | 0.0026 | D | D | N | D | N | N | N | D | D | 5/9 | D |
| 4:78910660 A>G | missense | NA | N | N | N | N | N | N | N | D | N | 1/9 | N |
| rs188481381 | missense | 4.00E-04 | D | N | N | N | N | N | N | D | N | 2/9 | N |
| rs1021174449 | missense | NA | D | D | N | D | D | N | D | D | D | 7/9 | D |
| 4:78910865 A>G | missense | NA | D | D | N | D | D | N | N | D | D | 6/9 | D |
| rs201726644 | missense | 2.00E-04 | D | D | N | D | D | N | N | D | D | 6/9 | D |
| **rs143310663** | missense | 0.0043 | D | D | N | D | D | N | N | D | D | 6/9 | D |
| 4:78911146 C>T | missense | NA | N | D | N | N | N | N | N | D | N | 2/9 | N |
| rs1734580253 | missense | 6.57E-06 | N | N | N | N | N | N | N | N | N | 0/9 | N |
| rs370160842 | missense | 7.88E-05 | D | N | N | N | N | N | N | N | N | 1/9 | N |
| rs1017952122 | missense | 1.32E-05 | D | D | N | D | D | N | N | N | D | 5/9 | D |
| rs532067029 | missense | 1.00E-04 | D | D | D | D | D | N | D | D | D | 8/9 | D |
| 4:78911564 A>G | missense | NA | D | D | N | D | D | N | N | D | D | 6/9 | D |
| rs776225825 | missense | 2.63E-05 | D | N | N | D | D | D | N | D | D | 6/9 | D |
| rs116710382 | missense | 0.0065 | D | N | N | N | N | N | N | NA | N | 1/8 | N |
| rs369782458 | missense | 7.23E-05 | D | D | N | N | N | N | N | N | N | 2/9 | N |
| rs138262352 C>T | stop gained | 0.0021 | NA | NA | NA | D | D | NA | NA | NA | N | 2/3 | D |
| rs138262352 C>G | missense | 6.00E-04 | D | D | N | D | D | N | N | D | D | 6/9 | D |
