# Supplementary material for: CYP2C19 Genotype is Associated with Citalopram Treatment Outcomes in a Real‐World Setting
Source: Clin Pharmacol Ther. 2026 Apr 14;120(2):412–9. doi: 10.1002/cpt.70285 (PMC13339076; doi:10.1002/cpt.70285)
Supplement: Supplementary file 2 — Figure S1. [file CPT-120-412-s002.docx]

**Supplementary Figure S1. Impact of CYP2C19 metabolizer phenotype on therapeutic failure and treatment resistance to citalopram in major depressive disorder (MDD).** UK Biobank participants with MDD who had a history of citalopram use were stratified by CYP2C19 metabolizer status defined as: poor metabolizer (PM, Null/Null), intermediate metabolizer (IM, *1/Null or *17/Null), normal metabolizer (NM, *1/*1), and ultrarapid metabolizer (UM, *1/*17 or *17/*17). Panels show the risk of (A) therapeutic failure and (B) treatment resistance within a prespecified follow-up window. Odds ratios relative to NM are presented with 95% confidence intervals. For visualization purposes, the x-axis was truncated at OR = 2.5, and confidence intervals exceeding this limit are indicated with arrows.

**Supplementary Figure S2. Hyponatremia while on citalopram by CYP2C19 genotype in the MDD cohort.** Odds ratios for hyponatremia (serum sodium <135 mmol/L) during citalopram exposure are shown for each CYP2C19 genotype. Squares indicate odds ratios and horizontal lines the 95% confidence intervals from Fisher’s exact tests versus *1/*1, displayed on a log scale. To assess a monotonic trend in risk across ordered genotypes, a logistic regression model with a single ordinal genotype term was fitted. The p-value for this trend term is reported on the plot.

**Supplementary Figure S3. Percentage of (A) between-class and (B) within-class switches from citalopram across CYP2C19 metabolizer groups in therapeutic failure and treatment resistance.** Bars show the proportion of patients whose first switch from citalopram was to a non-SSRI antidepressant (between-class) or to another SSRI (within-class).

**Supplementary Figure S4. Per-variant association signals in BMP2K.** The x-axis shows the odds ratio and the y-axis shows -log_10_(p) from Fisher’s exact test. Each dot denotes one or more variants with identical odds ratios and P-values. The petal count equals the number of variants with identical statistical test results. Only variants included in the BMP2K SKAT mask are displayed. The blue horizontal dashed line indicates p = 0.05.
